# *UHRF1* gene silencing inhibits cell proliferation and promotes cell apoptosis in human cervical squamous cell carcinoma CaSki cells

**DOI:** 10.1186/s13048-016-0253-8

**Published:** 2016-07-19

**Authors:** Ting-Ting Ge, Meng Yang, Zhuo Chen, Ge Lou, Tao Gu

**Affiliations:** Department of Gynecology, Harbin Medical University Cancer Hospital, No. 150 Haping Road, Nangang District, Harbin, 150040 Heilongjiang Province People’s Republic of China

**Keywords:** *UHRF1*, Cervical squamous cell carcinoma, CaSki cell line, Proliferation, Apoptosis

## Abstract

**Background:**

Up-regulation of UHRF1 has been observed in a variety of cancers and appears to serve as an independent prognostic factor.

**Objective:**

To explore the effect of *UHRF1* gene silencing on apoptosis and proliferation of cervical squamous cell carcinoma (CSCC) CaSki cells.

**Methods:**

This study consisted of 47 CSCC tissues and 40 normal cervical tissues. The CaSki cells were assigned into Blank group (CaSki cells not transfected), NC group (CaSki cells transfected with control siRNA), and UHRF1 Silence group (CaSki cells transfected with *UHRF1* siRNA). qRT-PCR and Western blot were used for *UHRF1* mRNA and protein expressions, CKK-8 assay for cell proliferation, flow cytometry for cell cycle and apoptosis, Western blot for expressions of apoptosis-related proteins. Nude mice tumor transplant experiment was performed.

**Results:**

UHRF1 exhibited higher mRNA and protein expressions in the CSCC tissues than normal cervical tissues (both *P* < 0.05). The cell proliferation ability in the UHRF1 Silence group was reduced when compared with the Blank group and the NC group, the cells at S-G2M stage in the UHRF1 Silence group were dropped when compared with the Blank group and the NC group (*P* < 0.05), while the cells at G0/G1 stage were elevated (*P* < 0.05), and the proportion of Annexin V positive cells in the UHRF1 Silence group was increased in comparison with the Blank group and the NC group (*P* < 0.05). Nude mice tumor transplant experiment indicated that the growth rate and weight of tumor in the Blank group and NC group was higher and heavier than the UHRF1 Silence group (*P* < 0.05).

**Conclusion:**

UHRF1 showed a high expression in CSCC and UHRF1 silencing can reduce proliferation and enhance apoptosis of the CaSki cells.

## Background

Cervical cancer is the third most commonly diagnosed gynecologic cancer and the fourth major cause of death-related cancer in females worldwide, accounting for 9 % of the total new diagnoses and 8 % of the total cancer-associated deaths among females in 2008 [1]. In China, there were 87,982 new patients diagnosed as cervical cancer and 23,375 deaths in 2011, and this disease has become one of the heaviest health burdens among Chinese women [2]. The major cause of cervical cancer refers to persistent human papillomavirus (HPV) infection, which is detected in 99 % of patients with cervical tumors [3]. Interestingly, cervical cancer develops in a multi-step process that implicates the transformation of normal cervical epithelium into preneoplastic cervical intraepithelial neoplasia (CIN) that ultimately progress to invasive cervical cancer cells [4]. Accounting for 70 ~ 80 % of cervical cancers, cervical squamous cell carcinoma (CSCC) consists of cells which are recognizably squamous but different from each other in term of growth pattern or cytological morphology [3]. The latest study reported there is no apparent symptom of early cervical cancer, while locally advanced disease may result in abnormal vaginal bleeding, pelvic pain, and dyspareunia [3]. Clearly, an improved understanding of the gene or molecular mechanisms via which pre-invasive lesions have the capability of invading the cervical stroma and eventually metastasis would exert significantly clinical influence.

UHRF1 [ubiquitin-like, containing plant homeodomain (PHD) and really interesting new gene (RING) finger domains 1], also known as ICBP90 or Np95, is a member of UHRF family and encode a 95-kDa nuclear protein of 793 amino acids, with a single open reading frame (ORF) [5]. It contains an N-terminal ubiquitylation-like domain, PHD, a SRA (SET and RING-associated) domain and a RING finger motif domain [6]. During the recent years, UHRF1 has received great concerns as a novel diagnostic marker of cancer [7]. UHRF1 is featured by a SRA domain (Set and Ring Associated) only found in the UHRF family, and up-regulation of UHRF1 has been observed in a variety of cancers, such as lung cancer and bladder cancer [8, 9]. Also, previous studies demonstrated that the expression level of UHRF1 is closely associated with clinical stage, metastatic and prognostic of bladder cancer and lung cancer [7, 9]. Furthermore, over-expression of UHRF1 is shown to contribute to worse survival of laryngeal squamous cell carcinoma (LSCC) and serve as an independent prognostic factor of LSCC [10]. Therefore, the present study aims at exploring the effects of silent *UHRF1* on proliferation and apoptosis of human CSCC Caski cells and to further study the related mechanism of cell apoptosis.

## Methods

### Tissue collection and cell culture

A total of 47 cases of CSCC tissues and 40 cases of normal cervical tissues resected from benign tumors were collected from Harbin Medical University Cancer Hospital from June 2013 to December 2015. The normal tissues were classified as the Control group and the CSCC tissues were as the CSCC group. The CSCC cell lines CaSki were obtained from cell bank of Chinese Academy of Sciences. The cells were cultured in 90 % RPMI1640 (Gibco BRL Co. Led., New York, USA) and 10 % Fetal bovine serum (FBS) (Gibco BRL Life Technologies Inc., Grand Island, New York, USA). *UHRF1* siRNA and control siRNA were purchased from Santa Cruz (sc-76805, Santa Cruz Biotechnology Inc., Santa Cruz, CA, USA). When the cells merged to 50 %, lentivirus diluted by medium solution were added for transfection. After 24 h, the virus was aspirated and normal solution was replaced. The cells were classified into 3 groups: the Blank group (CaSki cells untransfected), the negative control (NC) group (CaSki cells transfected with control siRNA) and the UHRF1 Silence group (CaSki cells transfected with *UHRF1* siRNA).

### Quantificational real-time polymerase chain reaction (qRT-PCR)

qRT-PCR was used for the detection of *UHRF1* expression level in 47 cases of CSCC tissues, 40 normal cervical tissues and differenttransfected CaSki cells (Blank group, NC group, and UHRF1 Silence group). The tissues were washed 3 times with phosphate buffered saline (PBS). After 72-h culture, the cells were washed again with PBS for 3 times. The total RNA extraction was according to RNAiso Plus reagent kit (Takara Bio Inc., Dalian, China) and reverse transcription was operated in conformity with PrimeScript RT reagent kit (Takara Bio Inc., Dalian, China). The SteponePlus PCR (Applied Biosystem, Foster City, CA, USA) system applied for fluorescence quantitative test was following: cDNA solution 1.6 ul, 2X SYBR GREEN Taq PCR MIX (Takara Bio Inc., Dalian, China) 5 ul, PCR upstream and downstream primers (10 uM, each 0.2 ul) and deuterium depleted water (DDW) 3 ul. Reaction condition was initial denaturation at 95 °C for 5 min, 95 °C 10 s, 58 °C 10 s, and 72 °C for 10 s, 60 cycles and extension at 72 °C for 10 min. The detection gene was *UHRF1* (Gene ID 29128) and internal reference gene was β-actin (Gene ID 11461). The primers (Invitrogen Biotechnology Co. Shanghai, China) were listed in Table 1. The experiment was repeated 3 times, and the average values were obtained.

**Table 1:** PCR primer sequences

Gene	Primer sequence
UHRF1	Forward: 5’- ACCAAGGTGGAGCCCTACAG -3’
	Reverse: 5’- CACTTTACTCAGGAACAACTGGAAC -3’
β-actin	Forward: 5’-CATCACGTACCAAACTTCAA-3’
	Reverse: 5’-CATCACAGTACCGGATTGC-3’

### Western blot assay

After transfection of 3 days, the cells were washed, added with Radio immunoprecipitation assay (RIPA) lysis buffer and protease inhibitor (Sigma, St. Louis, USA) for homogenate, and centrifuged at 12,000 × g at 4 °C for 10 min. After bicinchoninic acid (BCA) (Boi-rad Laboratories, Inc, Hercules, California, USA) detection of the protein concentration, the supernatant was reserved at −80 °C. Western blot applied 10 % sodium dodecyl sulfate polyacrylamide gel electropheresis (SDS-PAGE) gel. Each well was added with 20 ug protein samples. The primary anti-bodies were Rabbit anti-human-UHRF1, Caspase3, Caspase8, Caspase9, Bax and Bcl-2 (Santa Cruz Biotechnology Inc., Santa Cruz, CA, USA) and the cells were kept overnight at 4 °C. The secondary anti-body was POD-conjugated goat anti-rabbit (1:5000) and the cells were maintained at room temperature for 30 min, which was added with horseradish peroxidase (HRP) (Boi-rad Laboratories, Inc, Hercules, California, USA). Image Quant 350 and Image Quant TL-1 (GE Healthcare, Fairfield, CT, USA) were applied to analyze the results. The reference gene was β-actin. The experiment was repeated 3 times, and the average values were obtained.

### CCK-8 for cell proliferation

The CaSki cells were cultured in 96-hole plate at density of 1 × 10^5^ cells. Each group was designed with 4 duplicated wells. After 24-h culture, the cell proliferation was examined with Cell Counting Kit-8 (Thermo, USA) at 24, 48, 72 and 96 h. The procedures were as follows: After the aspiration of the original medium, the cells were added with CCK-8 solution. The medium was detected at 450 nm absorbance after 2-h culture. The experiment was repeated 3 times, and the average values were obtained.

### Flow cytometer

Propidium iodide (PI) was applied to analyze cell cycle, and Annexin V-FITC and PI (Abcam Co., Cambridge, MA, USA) were used for analysis of cell apoptosis rate. The CaSki cells were washed twice with PBS and added with 0.25 % pancreatin for digestion. After adjusting the density to 10^5^/ml, the cells were incubated with fluorescent antibody at room temperature for 30 min. The cells were precipitated and suspended after 200 × g centrifuging. Finally, the flow cytometer (BD Bioscience, San Jose, CA, USA) was applied for analysis. The experiment was repeated 3 times, and the average values were obtained.

### Establishment of tumor-bearing nude mice model

A total of 24 female nude mice of 4–6 weeks were (purchased from Shanghai Biomodel Organism Science & Technology Development Co., Ltd.) fed under sun protection factor (SPF) environment. The nude mice were injected with the CaSki cells in aseptic condition, and then were assigned into 3 groups (each group with 8 mice): the Blank group (injected with untransfected CaSki cell), the NC group (injected with CaSki cells transfecting control siRNA) and the UHRF1 Silence group (injected with CaSki cells transfecting *UHRF1* siRNA). The cell number injected was 1 × 10^6^ and the volume was 100 ul. Ten days later, the maximum and minimum diameter of the tumor was measured with vernier caliper every 5 days. The tumor size = maximum diameter × minimum diameter^2^. Thirty days later, the tumor tissue on the back of nude mice were taken out and measured the weight. The tissues were fixed with 10 % formaldehyde. After dehydration, the tissues were embedded with paraffin for staining analysis.

### Immunohistochemical staining

The tissues were washed with PBS for 3 times and fixed with 4 % paraformaldehyde. After embedding with paraffin and slicing, the tissues were performed immunohistochemical analysis. Firstly, the paraffin sections were dewaxed and rehydrated and treated with 3 % hydrogen peroxide for 15 min to block endogenous peroxidase activity. Then the tissues were heated with vapor for 30 min to restore antigen. The tissues were later blocked in PBS chamber containing 1 % bovine serum albumin (BSA). The primary anti-body was added for incubation at room temperature for 30 min and then maintained overnight at 4 °C. The primary antibody was rabbit polyclonal anti-proliferating cell nuclear antigen (PCNA) and UHRF1 (Abcam Co., Cambridge, MA, USA) and the slicess were kept overnight at 4 °C. The secondary antibody was POD-conjugated goat anti-rabbit (1 : 5000) and the slices were maintained at room temperature for 30 min, which was added with horseradish peroxidase (HRP) (Boi-rad Laboratories, Inc, Hercules, California, USA). The stained slices were pictured at 400 × amplification.

### Statistical methods

Data analysis was based upon the statistical package for the social sciences (SPSS) version 20.0 (SPSS Inc.; Chicago, IL, USA). Continuous data were displayed as mean ± standard deviation, and differences between two groups were examined by *t* test. And the differences among multiple groups were analyzed with repeated measurement of analysis of variance (ANOVA). Significance was illustrated by *P* < 0.05.

## Results

### *UHRF1* expression by qRT-PCR

**Fig. 1:**
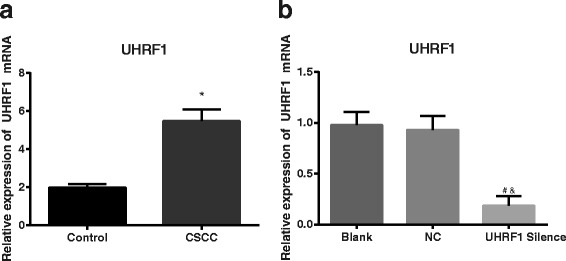
*UHRF1* mRNA expression detected by quantificational real-time polymerase chain reaction. **a** Comparison in *UHRF1* expression between CSCC tissues (*n* = 47) and normal cervical tissues (*n* = 40), revealing that *UHRF1* expression in CSCC tissues was much higher than that in normal cervical tissues; *, *P* < 0.05 compared with the Control group; **b** Comparison in *UHRF1* expression among CaSki cells of different transfection groups (*n* = 3), revealing that the UHRF1 Silence group had a significantly lower level of *UHRF1* mRNA expression than the Blank group and the NC group; #, *P* < 0.05 compared with the Blank group; &, *P* < 0.05 compared with the NC group; *UHRF1*, *ubiquitin-like, containing plant homeodomain (PHD) and really interesting new gene (RING) finger domains 1*; CSCC, cervical squamous cell carcinoma; NC, negative control

The fluorescent quantitative PCR indicated that the mRNA level of *UHRF1* was elevated in the tissues of the CSCC group in comparison with that in the Control group (*P* < 0.05). And the *UHRF1* mRNA expression in the UHRF1 Silence group was lower than those in the Blank group and the NC group (*P* < 0.05). No significant difference was found regarding *UHRF1* mRNA level between the Blank group and the NC group (*P* > 0.05, Fig. 1). The findings confirmed that *UHRF1* transcription level was promoted in the CSCC tissues when compared with the normal tissues and it could be inhibited by *UHRF1* siRNA in the CaSkin cell lines.

### UHRF1 expression by Western blot assay

**Fig. 2:**
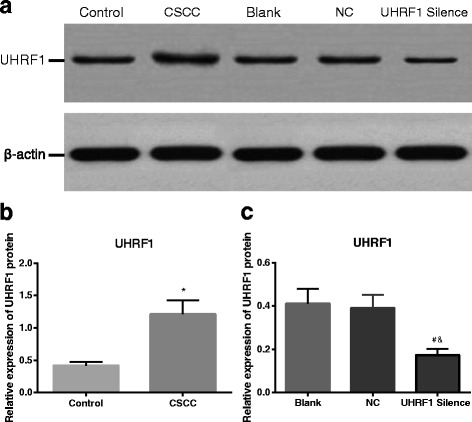
UHRF1 protein expression detected by Western blot. **a** Western blot map of CSCC tissues (*n* = 47), normal cervical tissues (*n* = 40) and CaSki cells in different transfection groups (*n* = 3); **b** Comparison of UHRF1 protein expression between CSCC tissues (*n* = 47) and normal cervical tissues (*n* = 40), showing that CSCC tissues had a significantly higher UHRF1 protein expression level than normal cervical tissues; *, *P* < 0.05 compared with the Control group; **c** Comparison of UHRF1 protein expression among CaSki cells in different transfection groups (*n* = 3), showing that the UHRF1 Silence group had a significantly lower level of UHRF1 protein expression than the Blank group and the NC group; #, *P* < 0.05 compared with the Blank group; &, *P* < 0.05 compared with the NC group; UHRF1, ubiquitin-like, containing plant homeodomain (PHD) and really interesting new gene (RING) finger domains 1; CSCC, cervical squamous cell carcinoma; NC, negative control

The results of Western blot revealed that UHRF1 protein expression in the CSCC group was higher than that in the Control group (*P* < 0.05). And the UHRF1 protein level in the UHRF1 Silence group was reduced when compared with the Blank group and the NC group (*P* < 0.05). No significant difference was found regarding UHRF1 protein level between the Blank group and the NC group (*P* > 0.05, Fig. 2). It was verified that UHRF1 expression was increased in the CSCC tissues when compared with the normal tissues and it could be inhibited by UHRF1 siRNA in the CaSkin cell lines.

### Cell proliferation by CCK-8

**Fig. 3:**
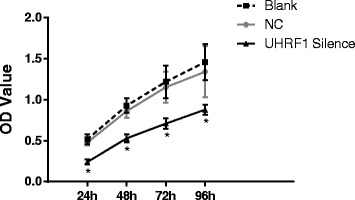
Cell proliferation detected by CCK-8. At each time point, the UHRF1 Silence group had a much lower proliferation ability than the Blank group and the NC group; *, *P* < 0.05 compared with the Control group, *n* = 3; UHRF1, ubiquitin-like, containing plant homeodomain (PHD) and really interesting new gene (RING) finger domains 1; NC, negative control

It was found that the cell proliferation ability in the UHRF1 Silence group was reduced when compared with the Blank group and the NC group (both *P* < 0.05). And there was no significant difference between the Blank group and the NC group (*P* > 0.05, Fig. 3). It was suggested that the proliferation ability of the CaSki cells was suppressed by *UHRF1* siRNA.

### Cell cycle by flow cytometer

**Fig. 4:**
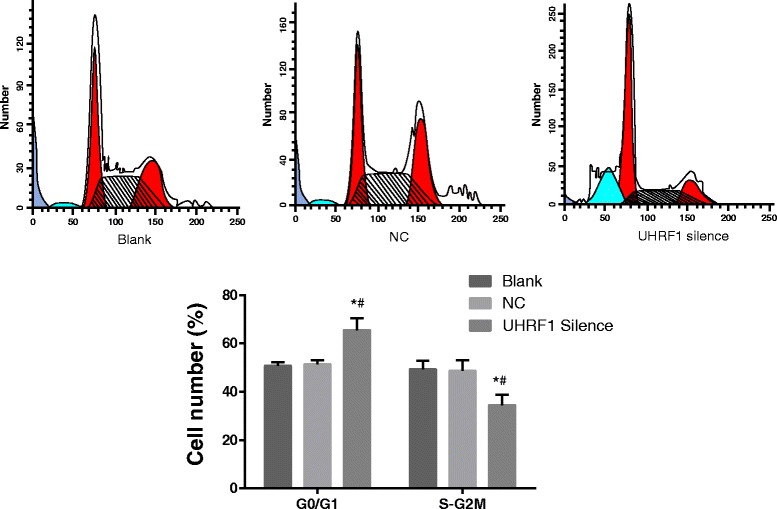
Cell cycle detected by flow cytometer. Compared with the Blank group and the NC group, the ratio of S-G2M phase cells in UHRF1 Silence group decreased significantly, while the ratio of G0/G1 phase cells increased obviously. The UHRF1 Silence group induced the apoptosis of CaSki cells, which could be substantiated by the appearance of sub-G1 peak in flow cytometric DNA histogram. The peak appeared ahead of the G0/G1 peak, and represented cell apoptosis. *, *P* < 0.05 compared with the Blank group; #, *P* < 0.05 compared with the NC group; *n* = 3; UHRF1, ubiquitin-like, containing plant homeodomain (PHD) and really interesting new gene (RING) finger domains 1; NC, negative control

The cells at S-G2M stage in the UHRF1 Silence group were dropped when compared with the Blank group and the NC group (*P* < 0.05, Fig. 4), while the cells at G0/G1 stage were elevated (*P* < 0.05). The UHRF1 Silence group induced the apoptosis of CaSki cells, which could be substantiated by the appearance of sub-G1 peak in the flow cytometric DNA histogram. The peak appeared ahead of the G0/G1 peak, and represented cell apoptosis. No significant difference existed between the Blank group and NC group (*P* > 0.05). Therefore, inhibiting U HRF1 was able to repress cell proliferation to stagnate the CSCC cells at G0/G1 stage.

### Cell apoptosis by flow cytometer

**Fig. 5:**
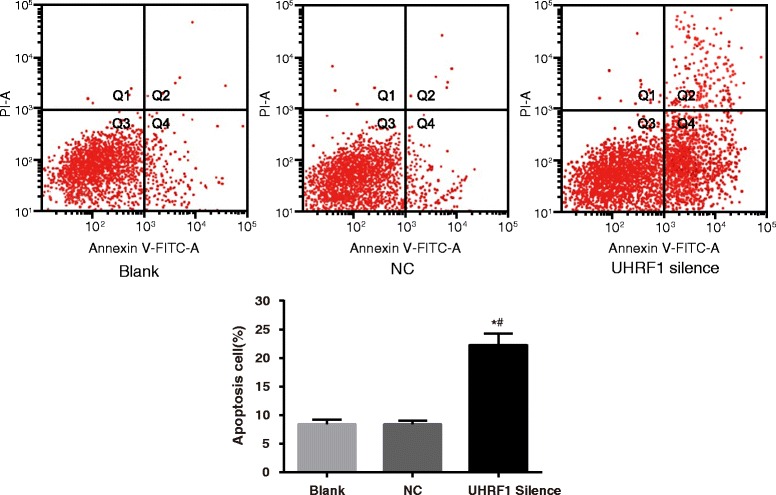
Cell apoptosis detected by flow cytometer. Annexin V + PI - represents early apoptosis cells (Q4), annexin V + PI + represents late apoptosis cells (Q2). The Annexin V-positive cells (early and late apoptosis cells) in the UHRF1 Silence group increased evidently as compared with the Blank group and the NC group, which indicated increased apoptosis cells. *, *P* < 0.05 compared with the Blank group; #, *P* < 0.05 compared with the NC group; *n* = 3; UHRF1, ubiquitin-like, containing plant homeodomain (PHD) and really interesting new gene (RING) finger domains 1; NC, negative control

It was found that the proportion of Annexin V positive cells (early apoptosis cells and late apoptosis cells) in the UHRF1 Silence group was increased in comparison with the Blank group and the NC group (*P* < 0.05, Fig. 5). No significant difference was found between the Blank group and the NC group (*P* > 0.05). Thus, the cell apoptosis rate was elevated when UHRF1 was inhibited.

### Apoptosis protein expressions by Western blot

**Fig. 6:**
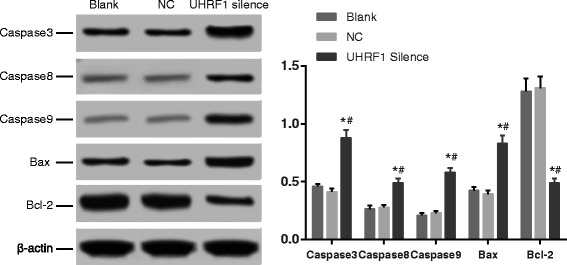
Apoptosis related protein expression detected by Western blot. Compared with the Blank group and the NC group, apoptosis-related proteins, namely Caspase3, Caspase8, Caspase9 and Bax, had a much higher level in the UHRF1 Silence group, while anti-apoptotic protein (Bcl-2) had a much lower level. *, *P* < 0.05 compared with the Blank group; #, *P* < 0.05 compared with the NC group, *n* = 3; UHRF1, ubiquitin-like, containing plant homeodomain (PHD) and really interesting new gene (RING) finger domains 1; NC, negative control

The expressions of the apoptosis proteins Caspase3, Caspase8, Caspase9 and Bax and anti-apoptosis protein Bcl-2 in the Blank group were not significantly different from those in the NC group (all *P* > 0.05). However, the expressions of above-mentioned apoptosis proteins in the UHRF1 Silence were elevated, while the anti-apoptosis protein was reduced (all *P* < 0.05, Fig. 6), which further confirmed that the inhibition of UHRF1 promoted cell apoptosis of the CaSki cells.

### Cell malignant transformation in tumor-bearing nude mice model

**Fig. 7:**
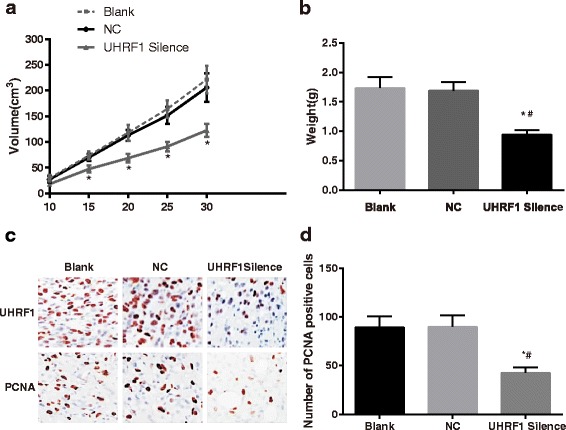
Tumor-bearing nude mice model experiment. **a** growth curve of tumor volume. The tumor growth speed in the *UHRF1* Silence group was much slower than that in the Blank group and NC group; **b** tumor weight. The tumor weight in the UHRF1 Silence group was much lower than that in the Blank group and the NC group; **c** immunohistochemical staining of tumor sections; **d** the number of PCNA-positive cells. The number of UHRF1-positive and PCNA-positive cells in the UHRF1 Silence group was much smaller than that in the Blank group and NC group. *, *P* < 0.05 compared with the NC group; #, *P* < 0.05 compared with the NC group; UHRF1, ubiquitin-like, containing plant homeodomain (PHD) and really interesting new gene (RING) finger domains 1; NC, negative control; PCNA, proliferating cell nuclear antigen

The experiment indicated that the tumor growth speed in the UHRF1 Silence group was slower than that in the Blank group and NC group (*P* < 0.05, Fig. 7a). The tumor in the UHRF1 Silence group was also thinner than that in the Blank group and the NC group (*P* < 0.05, Fig. 7b). The slice staining results suggested that the UHRF1 and PCNA positive cells in the UHRF1 Silence group were less than the Blank group and the NC group (Fig. 7c), which further indicated that in the tumor-bearing nude mice model, the cell proliferation in the UHRF1 Silence group were significantly lower than the Blank group and the NC group. Therefore, the silent UHRF1 could inhibit the cell malignant transformation in the CaSki cells of the nude mice.

## Discussion

As a leading cause of mortality in women, cervical cancer still exhibits unclear molecular mechanisms [4]. Despite of the significant advances including surgical techniques, radiotherapy and chemotherapy, there are still nearly 30 % of patients diagnosed with invasive cervical carcinoma that passes away due to residual or recurrent disease [11]. However, increasing evidence indicates that the expression or overexpression of UHRF1 is associated with a poor prognosis in a variety of cancer types [7, 9]. Therefore, the aim of the current study was to evaluate the potential association between UHRF1 and CSCC. Consequently, UHRF1 is over-expressed in CSCC and silencing UHRF1 inhibits proliferation and promotes apopyosis of the CSCC CaSKi cells.

In this study, we firstly assessed the expression level of UHRF1 in the CSCC tissues. We found that UHRF1 mRNA and protein were highly regulated in CSCC tissues in comparison to the normal cervical tissue, suggesting that UHRF1 participates in the development and evolvement of CSCC. Similarly, Yan et al. supported that UHRF1 has been considered to be an oncogene which is involved in tumorigenesis in ovarian cancer [6]. To our knowledge, UHRF1 can form a complex with DNA methyltransferase 1 (DNMT1) as well as histone deacetylase 1 (HDAC1) and can restrict the expression of some tumor suppressor genes through its SRA domain, such as p16 INK4A, hMLH1, BRCA1 and RB1 [8]. According to former studies, high expression of UHRF1 was also detected in lung cancer cells, particularly in prostate cancer and breast cancer and it seem that the intensity of its over-expression was linked to the clinical stage of the cancer [12, 13]. UHRF1 has recently been shown to play an important role in DNA methylation maintenance [14]. It has the ability to trigger the collection of DNMT and histone deacetylace-1 by binding hemimethylated DNA via its SET- and RING-associated domain [15–17]. Krifa et al. and Alhosin et al. have proved that UHRF1, as an oncogene protein, has the potential to suppress tumor suppressor gene expression such as p16INK4A, hMLH1, BRCA1 along with RB1 through binding to methylated DNA and recruiting the DNMT1 [8, 18]. Also, several studies indicate that over-expression of UHRF1 plays a part in the changed DNA methylation patterns and the establishment of aberrant histone code [19, 20].

Importantly, UHRF1 is essential for cell proliferation, and depleting UHRF1 may inhibit cell proliferation and lead to cell death. The results indicated that silencing UHRF1 leads to a decrease in the proliferation of CaSki cells and the apoptosis of CaSKi increases (the activity of Caspase3, Caspase8, Caspase9, Bax apoptosis-related protein increase). This is consistent with previous studies showing that down-regulation of UHRF1 leads to cell growth inhibition [19, 20]. According to the research of Li et al., up-regulated UHRF1 might increase cyclin D1 activityand Bax downregulation, resulting in G1 shortage and inhibition of apoptosis and subsequent promotion of cell proliferation respectively [5]. As it was reported, the *UHRF1* gene, its expression is associated with increased cell proliferation, is a target for the transcriptional activator E2F1 [21]. The results of nude mouse experiment indicated that tumor grow more slowly in UHRF1 silence group, implying that silencing UHRF1 can suppress the tumorigenesis of CaSKi cells in nude mouse. Moreover, UHRF1-knockout mouse embryonic stem cells exhibit significant loss of genomic methylation [14].

Furthermore, in our study, the proliferation of CaSKi was prohibited when UHRF1 was down-regulating and cells are stay at G0/G1 stage. Alteration in UHRF1 expression is correlated with the degree of the lung cancer aggressiveness and it was detected in 50 % of the patients in an early clinical stage [7]. Daskalos and his colleagues reported that UHRF1 is a prominent epigenetic switch that controls cell cycle in lung carcinoma due to its ability to smiantain the transcriptional silencing of tumor suppressor genes through sustaining their promoters in a hypermethylated condition [21]. UHRF1 mRNA and protein fluctuate with the cell cycle [22, 23], inactivation or loss of *UHRF1* blocks S-phase entry [24], and zebrafish with a depletion-of-function mutation in *UHRF1* have reduced cell proliferation and promoted cell apoptosis in hepatocyte [25]. These data suggests that up-regulating cancer cells of *UHRF1* may result in cell death.

## Conclusions

In conclusion, our study uncovered that UHRF1 overexpressed in CSCC and silent *UHRF1* gene can reduce tumorigenicity of the CaSki cells by decreasing its proliferation capacity and making it stagnate at G0 and G1 phases as well as accelerating its apoptosis. Altogether, our data, combined with existing evidence provides a basis for investigating *UHRF1* as a possible therapeutic target in human cancer.

## Abbreviations

ANOVA, analysis of variance; BCA, bicinchoninic acid; BSA, bovine serum albumin; CIN, cervical intraepithelial neoplasia; CSCC, cervical squamous cell carcinoma; DDW, deuterium depleted water; DNMT1, DNA methyltransferase 1; FBS, fetal bovine serum; HDAC1, histone deacetylase 1; HPV, human papillomavirus; HRP, horseradish peroxidase; LSCC, laryngeal squamous cell carcinoma; NC, negative control; ORF, open reading frame; PBS, phosphate buffered saline; PCNA, proliferating cell nuclear antigen; PI, propidium iodide; qRT-PCR, quantificational real-time polymerase chain reaction; RIPA, radio immunoprecipitation assay; SDS-PAGE, sodium dodecyl sulfate polyacrylamide gel electropheresis; SPF, sun protection factor; SPSS, statistical package for the social sciences; SRA, Set and Ring-associated; UHRF1, ubiquitin-like, containing plant homeodomain and really interesting new gene finger domains 1
